# Evaluation of the growth, adaption, and ecosystem services of two potentially-introduced urban tree species in Guangzhou under drought stress

**DOI:** 10.1038/s41598-023-30782-x

**Published:** 2023-03-02

**Authors:** Muhan Zhang, Yuan Ni, Mingwei Li

**Affiliations:** 1 Anhui Institute of Modern Agricultural Engineering, Hefei, China; 2 Guangdong Eco-Engineering Polytechnic, Guangzhou, China

**Keywords:** Climate-change ecology, Urban ecology

## Abstract

Under rapid urbanization and agglomeration of population, cities are facing various environmental challenges. As urban forests play a crucial role in mitigating native environmental problems and providing ecosystem services, cities might enhance their urban forest construction through multiple approaches, of which the introduction of exotic tree species could be an effective way. Under the background of constructing a high-quality forest city, Guangzhou was considering introducing a series of exotic tree species to improve the local urban greening, among which *Tilia cordata* Mill. and *Tilia tomentosa* Moench became the potential objects. As Guangzhou was reported to experience higher temperatures with less precipitation and face drought events with increasing frequency and intensity, whether the two tree species could survive in the dry environment required to be investigated profoundly. Thus, we launched a drought-simulation experiment and measured their above- and below-ground growth in 2020. In addition, their ecosystem services were also simulated and evaluated for their future adaption. Furthermore, a congeneric native tree species *Tilia miqueliana* Maxim was also measured in the same experiment as a comparison. Our results showed that *Tilia miqueliana* exhibited moderate patterns of growth and advantages in evapotranspiration and cooling. Besides, its investment in root development at horizontal level could account for its special strategy against drought stress. *Tilia tomentosa*’s vigorous root growth could be the most positive behavior of coping with water deficit, which explained its maintenance of carbon fixation and implied a well adaption. *Tilia cordata* showed a complete decrease in above- and below-ground growth, especially for its fine root biomass. In addition, its ecosystem services were significantly reduced, reflecting a comprehensive failure when it faced a long-term scarcity of water. Therefore, it was necessary to supply sufficient water and under-ground space for their living in Guangzhou, especially for *Tilia cordata*. In the future, long-time observation of their growth under different stresses can be practical approaches to amplify their multiple ecosystem services.

## Introduction

Rapid urbanization and agglomeration of population are exposing cities to various environmental challenges such as atmospheric contaminations, pollution caused by solid wastes, and heat island effect^1–3^. To mitigate negative problems and improve urban environment, urban forests and trees are considered to play an essential role through supplying multiple ecosystem services^4,5^. Therefore, the construction and conservation of urban forests and greening have drawn worldwide attention from diverse perspectives. For example, Zhao et al. proposed specific recommendations regarding how to improve the quality of urban forest construction from ecological and landscape aspects in southern China^6^. Bonilla-Duarte et al. studied urban forest sustainability in residential areas and suggested that forest planning in cities should consider tree species, the design, and the structure of spatial arrangements^7^. Furthermore, local voices from residents should also be encouraged within the design of urban forest construction in the future^8^.

Among various approaches to urban forest construction, the introduction of exotic tree species could be an effective way which could promote biodiversity, improve regional landscape values, and enrich forest structures at different scales^9,10^. Importantly, the adaption of introduced tree species should be comprehensively evaluated in the objected areas, otherwise, the unfitted tree species might result in high mortalities with economic losses, or even regional instability of communities accompanying degenerations of local ecosystem^11,12^. For example, Ennos et al. suggested that novel exotic tree species bring elevated economic, biosecurity and ecological risks and required comprehensive risk assessment across forestry sectors^13^. Dodet and Collet highlighted that complementary management strategies should be established to control exotic tree species at different stages of the invasion process^14^. Hence, comprehensive information on the above- and below-ground growth of potentially introduced tree species in objected cities would contribute enormously to a better understanding of their future adaption, based on which forestry administration departments could make specific policies for their conservation and management.

For the purpose of being a national forest city, Guangzhou took remarkable actions to increase its urban forest cover, of which the introduction of exotic tree species was adopted to enhance the local greening^6^. However, information on the adaption of some potentially- or newly-introduced tree species remained scarce. In addition, Guangzhou was experiencing higher average temperatures and less precipitation for the past years^15^, and it was predicted to encounter drought events with increasing intensities and frequencies under the global change^16^. Under this background, the survival of exotic tree species might confront extreme drought stresses since they were planted. As water availability played a key role in supporting tree growth, many researchers pointed out that drought stress would restrict urban trees’ growth and their ecosystem services^17^. For example, some researchers found that the photosynthetic productivity and tree growth would be severely affected by water scarcity^18^. Moser also verified that a long drought period would diminish the overall growth of some urban tree species in Germany, following a tremendous loss of carbon fixation. Thus, it was necessary to take exotic tree species’ drought tolerances into account, which helped to provide sufficient knowledge regarding their future adaption in Guangzhou.

Roots, especially fine roots, are the primary pathway for water and nutrient uptake^19^. Particularly under drought stress, trees could dynamically adjust their investment in root growth from different perspectives, such as increasing the growth at vertical or horizontal levels, and accelerating root turnover rates^20,21^. For urban trees, the below-ground development patterns could reflect tree’s adaption adequately as this could be the most efficient and practical way of coping with harsh surroundings and restricted water supply^22^. Therefore, to evaluate the adaption of two potentially-introduced tree species in Guangzhou, we launched a drought-simulation experiment and measured their above- and below-ground growth, including the development of tree height, diameter in breast height, crown areas, and fine root biomass at different directions. Meanwhile, their primary ecosystem services were also simulated and compared between the controlled and drought-simulation groups. Additionally, a congeneric native tree species was also measured in the same experiment which was regarded as a comparison. Based on these information, we aimed to answer: (1) how do the two tree species respond to water shortage in terms of above-ground growth? (2) What were their particular strategies within their fine root systems under water deficit? (3) What were the impacted extents of their ecosystem services in an arid environment? (4) If they were planted in Guangzhou, what special management was appropriate for them?

## Materials and methods

### Study site, tree selections, and drought-simulation experiment

This research was performed in Guangzhou (22°26′-23°56′N, 112°57′-114°03′E), which is a core city located in subtropical zones. With an area of 7434.4 km^2^ and a population of 18.87 million, Guangzhou’s urbanization rate has reached 86.46%. To cope with multiple environmental challenges, several urban-forest nurseries were established to cultivate and introduce various tree species. Among them, we selected the one in Tianhe District as our study site. This nursery was not only abundant with native and exotic tree species but also equipped with similar edatope in cities, which was ideal for our research.

*Tilia cordata* Mill. (*Tc*) and *Tilia tomentosa* Moench (*Tt*), originating from the west of Britain and southeast of Europe, were common urban tree species planted in European cities. Based on their performance in providing ecological and landscape functions, these two tree species were considered to be introduced for urban greening. Therefore, *Tilia cordata* Mill. (*Tc*) and *Tilia tomentosa* Moench (*Tt*) were selected as our objectives, which were investigated for their growth and ecosystem services to evaluate their adaption in Guangzhou. In addition, a native tree species *Tilia miqueliana* Maxim (*Tm*) was also implemented concurrent measurement as a comparison.

**Figure 1:**
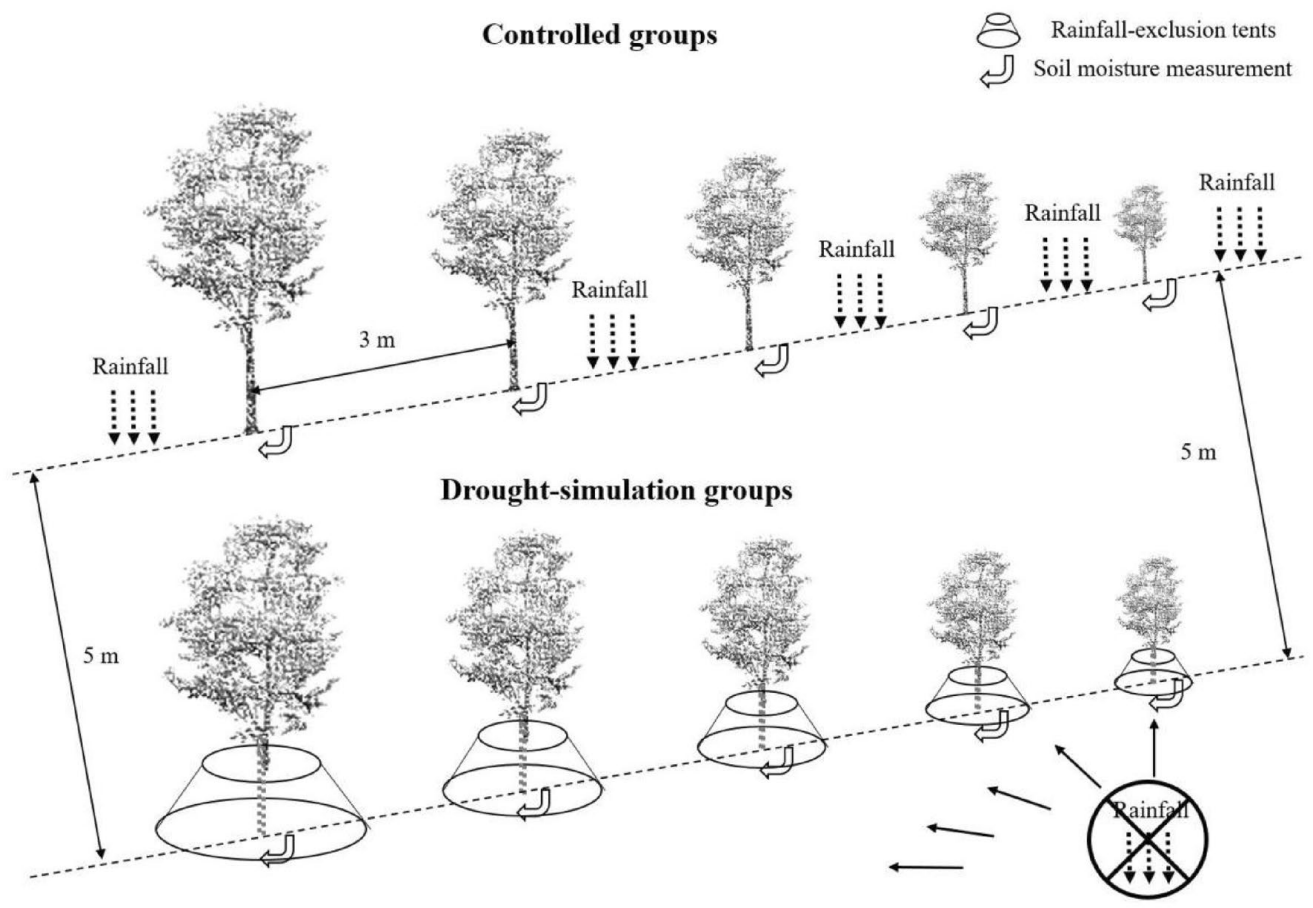
Schematic diagram of the drought simulation experiment for the three surveyed tree species.

For each of the three surveyed tree species, ten trees with a diameter at breast height (DBH) around 5.5 cm and tree height around 2.5 m were chosen for our experiment, which were thought to possess similar initial statuses. To investigate the impact of drought on the growth and ecosystem services of the three selected tree species, a controlled experiment was launched from January to December in 2020. For each tree species, five trees were planted in the common environment as the controlled group, while the other five trees were under the precipitation-exclusion installation (PEI) as the drought-simulation group. Consisting of several water-proof tents, PEI was adequately large and could completely prevent trees from obtaining rainfalls, which created a precipitation-exclusive environment to simulate an enduring drought event within the whole research period (Fig. 1).

### Environmental monitoring systems

Climatic data were sampled every 10 min with a weather station (WP3103 mesoscale automatic weather station, China) located at an unshaded site in the nursery. The data were stored in the logger and copied to our laboratory to produce daily or monthly data. All the climatic variables, including photosynthetically active radiation (PAR, µmol m^-2^ s^-1^), wind speed (m s^-1^), precipitation (mm), and air temperature (°C) were calculated from January to December in 2020.

For volumetric soil water content (%; VWC), the HOBO MX2307 system (Onetemp, Adelaide, Australia), placed in a shaded box in the nursery, was applied for all the three tree species from both the controlled and drought-simulation groups. For each individual tree, the sensing probe was inserted horizontally at the depths of 30 cm and located 20 cm in the northern direction from the tree stems. Based on the daily readings, monthly means were calculated from January to December in 2020.

### Measurement of above-ground growth

To investigate the above-ground growth of the three tree species from both the controlled and drought-simulation groups, their DBH (diameter at breast height, cm), tree height (m), and LAI (leaf area index) were measured at the beginning of each month in 2020. DBH was measured with the help of a caliper (Altraco Inc., Sausalito, California, USA), and their tree heights were measured using a standard tape. The crown analytical instrument CI-110 (Camas, Washington State, USA) was used to capture an accurate image of tree crowns and calculate LAI. Sufficient numbers of points were measured and recorded to describe each tree’s average crown shape. The software FV2200 (LICOR Biosciences, Lincoln, NE) helped compute each tree’s crown width and crown area.

### Measurement of below-ground growth

Fine root coring campaigns were launched for all the trees of the three tree species from both the controlled and drought treatment groups every three to four months, i.e., in February, May, September, and December. Although the coring campaign might damage part of the roots, the fine roots obtained each time were a mere portion of the whole root system, not affecting the general development of trees’ underground processes. For every individual tree, two 30-cm soil cores were applied in each direction of north, south, east, and west, of which one was located at 20 cm to the trunk (paracentral roots) and the other one was located at 40 cm (outer roots). In addition, the soil samples were evenly divided into three horizons which were 0–10 cm (shallow layer), 10–20 cm (middle layer), and 20–30 cm (deep layer). Then a sieve with 2-mm mesh size was used to filter all the fine roots. The fine roots were washed carefully to remove the adherent soils and dried in an oven at 65 ℃ for 72 h. Finally, all the samples were weighed using a balance with an accuracy of four decimal places to obtain the dry weight. The fine root biomass at different depths was calculated using the dry weight divided by the cross-sectional area of the auger^20^.

### Model’s simulation of ecosystem services

The process-based model *City-Tree* was used to predict the ecosystem services of the three tree species from both the controlled and drought-simulation groups^23^. The model required the data of tree growth parameters including tree height, DBH, and crown area together with environmental conditions such as edaphic and climatic data^24^. In this research, cooling, evapotranspiration and CO_2_ fixation of the three surveyed tree species in the controlled and drought-treatment groups were simulated at the end of 2020.

The actual evapotranspiration et_a_ was calculated from the potential evapotranspiration using f_etp_[t], Tilia’s factors f_etp_[t], and the reduction factor f_red_:

$${\mathrm{et}}_{\mathrm{a}}={\mathrm{f}}_{\mathrm{red}}*{\mathrm{f}}_{\mathrm{etp}}\left[\mathrm{t}\right]*{\mathrm{et}}_{\mathrm{p}}$$

The process of tree’s evapotranspiration (et_p_) was calculated on the basis of SVAT algorithm together with Penman formula in the module on water balance as below:

$${\mathrm{et}}_{\mathrm{p}}=\left[\mathrm{s }/ \left(\mathrm{s}+\upgamma \right)\right]*\left({\mathrm{r}}_{\mathrm{s}}-{\mathrm{r}}_{\mathrm{L}}\right) /\mathrm{ L}+\left[1-\mathrm{s }/ \left(\mathrm{s}+\upgamma \right)\right]*{\mathrm{e}}_{\mathrm{s}}*\mathrm{f }\left({\mathrm{v}}_{\mathrm{u}}\right)$$

with γ: psychrometric constant in hPa K^−1^; s: the slope of the saturation vapour pressure curve in hPa K^−1^; r_s_: short wave radiation balance in W m^−2^; r_L_: long-wave radiation balance in W m^−2^; L: specific evaporation heat in W m^−2^ mm^−1^ d; e_s_: saturation deficit in hPa; f (v_u_): ventilation function with v_u_ being the daily average wind speed in m s^−1^.

Within the module cooling, the energy needed for the transition of water from liquid to gaseous phase was calculated based on the crown area (CA) and the transpiration et_a_ sum:

$${\mathrm{E}}_{\mathrm{A}}= {\mathrm{et}}_{\mathrm{a}}*\mathrm{CA}-\left({\mathrm{L}}_{\mathrm{O}}* -0.00242*\mathrm{temp}\right) / {\mathrm{f}}_{\mathrm{con}}$$

with E_A_: energy released by a tree through transpiration (kWh tree^-1^), L_O_: energy needed for the transition of the 1 kg of water from the liquid to gaseous phase = 2.498 MJ (kgH_2_O)^-1^ and temp = temperature in ℃, f_con_: 0.5.

The calculation of new assimilation in the module of photosynthesis and respiration was on the basis of the approach of Haxeltine and Prenticem^25^. The model assumed that 50% of the incoming short-wave radiation is photosynthetic active radiation (PAR). Using the LAI and a light extinction factor of 0.5, the radiation amount of 1 m^2^ leaf area can be estimated based on an exponential function according to the Lambert–Beer law. This way, the gross assimilation per m^2^ leaf area as the daily mean of the month can be derived from:

$${\text{A}} = {\text{d}}*{{\left[ {\left( {{\text{J}}_{{\text{p}}} + {\text{J}}_{{\text{r}}} - {\text{sqrt}} \left( {\left( {{\text{J}}_{{\text{P}}} + {\text{J}}_{{\text{r}}} } \right)^{2} - 4*\uptheta *{\text{J}}_{{\text{p}}} *{\text{J}}_{{\text{r}}} } \right)} \right)} \right]} \mathord{\left/ {\vphantom {{\left[ {\left( {{\text{J}}_{{\text{p}}} + {\text{J}}_{{\text{r}}} - {\text{sqrt}} \left( {\left( {{\text{J}}_{{\text{P}}} + {\text{J}}_{{\text{r}}} } \right)^{2} - 4*\uptheta *{\text{J}}_{{\text{p}}} *{\text{J}}_{{\text{r}}} } \right)} \right)} \right]} {\left( {2*\uptheta } \right)}}} \right. \kern-0pt} {\left( {2*\uptheta } \right)}}$$

with A: gross assimilation [g C m^−2^ d^−1^]; d: mean day length of the month [h]; J_p_: reaction of photosynthesis on absorbed photosynthetic radiation [g C m^−2^ h^−1^]; J_r_: rubisco limited rate of photosynthesis [g C m^−2^ h^−1^]; θ: form factor = 0.7.

J_p_ was defined as a function of the photosynthetic active radiation PAR in mol m^−2^ h^−1^ and the efficiency of carbon fixation per absorbed PAR [g C mol^−1^].

$${\text{J}}_{{\text{p}}} = {\text{c}}_{{\text{p}}} {\text{*PAR}}$$

$${\text{c}}_{{\text{p}}} = \alpha *\left( {{\text{p}}_{{{\text{ci}}}} - {\text{r}}} \right){ /}\left( {{\text{p}}_{{{\text{ci}}}} - {\text{r}}} \right)*\gamma *{\text{m}}_{{{\text{co}}_{2} }} *{\text{i}}\left[ {\text{t}} \right]$$

with α: intrinsic quantum efficiency for CO_2_ uptake = 0.08; p_ci_: partial pressure of the internal CO_2_ [Pa]; r: CO_2_ compensation point [Pa]; ϒ: species dependent adjustment function for tree age; m _CO2_: molecular mass of C = 12.0 g mol^−1^; i[t]: influence of temperature on efficiency.

Net assimilation A_N_ [g C m^−2^ d^−1^] was then derived from the gross assimilation A and the dark respiration R_d_ by:

$${\text{A}}_{{\text{N}}} = {\text{A}} - {\text{R}}_{{\text{d}}}$$

$${\text{R}}_{{\text{d}}} =\upbeta *{\text{V}}_{{\text{m}}}$$

where V_m_ was calculated as:

$${\text{V}}_{{\text{m}}} = {1 \mathord{\left/ {\vphantom {1 \upbeta }} \right. \kern-0pt} \upbeta } * {{{\text{c}}_{{\text{p}}} } \mathord{\left/ {\vphantom {{{\text{c}}_{{\text{p}}} } {{\text{c}}_{{\text{r}}} * {\text{PAR}} * \left[ {\left( {2\uptheta - 1} \right) * \beta * {{\text{d}} \mathord{\left/ {\vphantom {{\text{d}} {{\text{d}}_{\max } }}} \right. \kern-0pt} {{\text{d}}_{\max } }} - \left( {2\uptheta *\upbeta *{{\text{d}} \mathord{\left/ {\vphantom {{\text{d}} {{\text{d}}_{\max } }}} \right. \kern-0pt} {{\text{d}}_{\max } }} - {\text{c}}_{{\text{r}}} } \right)*\varsigma } \right]}}} \right. \kern-0pt} {{\text{c}}_{{\text{r}}} * {\text{PAR}} * \left[ {\left( {2\theta - 1} \right) * \beta * {{\text{d}} \mathord{\left/ {\vphantom {{\text{d}} {{\text{d}}_{\max } }}} \right. \kern-0pt} {{\text{d}}_{\max } }} - \left( {2\theta *\upbeta *{{\text{d}} \mathord{\left/ {\vphantom {{\text{d}} {{\text{d}}_{\max } }}} \right. \kern-0pt} {{\text{d}}_{\max } }} - {\text{c}}_{{\text{r}}} } \right)*\varsigma } \right]}}$$

By multiplying A_N_, the number of days and the total leaf area, the entire monthly net assimilation of the tree can be obtained. In this study, we assumed a fixed share of 50% as respiration based on the gross primary production that the resulting net primary production NPP was transformed in the content of fixed carbon by multiplying the value with the carbon conversion factor 0.5^24^.

$${\mathrm{Carbon}}_{\mathrm{fix}}=0.5*\mathrm{NPP}$$

### Statistical analyses

The software package R was used for statistical analysis. To investigate the differences between means, two-sampled t-test and analysis of variance (ANOVA) with Tukey’s HSD (honestly significant difference) test were used. All the cases, the means were reported as significant when *P* < 0.05. Where necessary, data were log or power transformed in order to correct for data displaying heteroscedasticity.

## Results

### Climatic and edaphic variables in the study site

**Figure 2:**
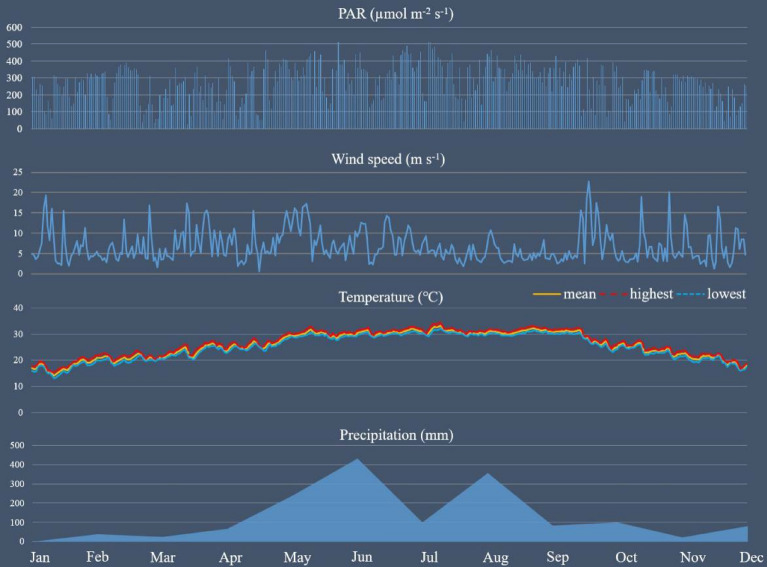
Monthly climate variables including PAR (µmol m^-2^ s^-1^), precipitation (mm), wind speed (m s^-1^), highest temperature (°C), mean temperature (°C), and lowest temperature (°C) in the study site from January to December in 2020.

With an average PAR of 272.81 µmol m^-2^ s^-1^, an average temperature of 25.82 °C, and annual precipitation of 1543.1 mm, the year 2020 in Guangzhou was very sunny and warm (Fig. 2). Compared to the historic records of the past 30 years (average temperature of 23.1 °C and annual precipitation of 1603 mm, National Meteorological Information Center of China), Guangzhou displayed a rising tendency of air temperature but a decreasing trend of annual precipitation. In addition, the highest temperature reached 34.52 °C in July, which was higher than the year 2019 (34.49 °C). In terms of wind speed, the mean value was 6.84 m s^-1^ and fluctuated between 0.52 m s^-1^ and 22.72 m s^-1^. Besides, the majority of the precipitation came in June and August (431.9 mm and 356.1 mm), while the other months exhibited relatively lower values. To sum up, the climate in Guangzhou was verified to become drier and warmer.

Regarding soil moisture, the controlled group exhibited significant differences from the drought-simulation groups in most of the months, mainly occurring after April (Table 1). In the controlled groups of all the three tree species, the volumetric soil water content (VWC) reached peaks in June (*Tc*-con: 0.34 ± 0.02; *Tt*-con: 0.37 ± 0.00; *Tm*-con: 0.36 ± 0.02), which was consistent with the highest values of precipitation in this month. While in the other months, VWC showed no distinct differences and fluctuated around 0.25. In the drought-simulation groups, VWC of all the three tree species had evident descending trends since the installation of the rainfall-exclusion tents, especially from April, significant decreases were observed, e.g., (*Tc*-con: 0.24 ± 0.04; *Tc*-dry: 0.18 ± 0.03). At the end of 2020, VWC from the three drought-simulation groups were 0.07 ± 0.01(*Tc*-dry), 0.08 ± 0.01 (*Tt*-dry), and 0.08 ± 0.01 (*Tm*-dry), which was lower than the controlled groups (*Tc*-con: 0.25 ± 0.02; *Tt*-con: 0.26 ± 0.01; *Tm*-con: 0.25 ± 0.01), indicating the overall success of the drought simulation.

**Table 1:** Monthly volumetric soil water content (VWC with standard deviation) at 20-cm soil depth of both the control and drought-simulation groups from January to December in 2020.

Month	VWC ± SD in all the groups					
	*Tc*-control	*Tc*-dry	*Tt*-control	*Tt*-dry	*Tm*-control	*Tm*-dry
Jan	0.26 ± 0.02	0.25 ± 0.04	0.26 ± 0.02	0.26 ± 0.04	0.25 ± 0.02	0.25 ± 0.04
Feb	0.27 ± 0.04	0.22 ± 0.03	0.28 ± 0.02	0.24 ± 0.03	0.27 ± 0.02	0.23 ± 0.03
Mar	0.26 ± 0.03	0.21 ± 0.04	0.28 ± 0.02	0.22 ± 0.02	0.26 ± 0.02	0.23 ± 0.03
Apr	0.24 ± 0.04	0.18 ± 0.03*	0.26 ± 0.02	0.21 ± 0.03	0.24 ± 0.02	0.20 ± 0.03
May	0.31 ± 0.02	0.13 ± 0.03*	0.31 ± 0.03	0.19 ± 0.02*	0.31 ± 0.02	0.16 ± 0.02*
Jun	0.34 ± 0.02	0.12 ± 0.01*	0.37 ± 0.00	0.15 ± 0.02*	0.36 ± 0.02	0.15 ± 0.02*
Jul	0.26 ± 0.07	0.12 ± 0.02*	0.30 ± 0.01	0.10 ± 0.02*	0.29 ± 0.02	0.14 ± 0.02*
Aug	0.30 ± 0.04	0.11 ± 0.01*	0.33 ± 0.02	0.09 ± 0.01*	0.33 ± 0.02	0.10 ± 0.01*
Sep	0.26 ± 0.04	0.09 ± 0.02*	0.25 ± 0.02	0.09 ± 0.01*	0.27 ± 0.02	0.09 ± 0.01*
Oct	0.27 ± 0.03	0.09 ± 0.01*	0.28 ± 0.01	0.09 ± 0.01*	0.28 ± 0.01	0.08 ± 0.01*
Nov	0.26 ± 0.03	0.08 ± 0.01*	0.25 ± 0.01	0.08 ± 0.01*	0.26 ± 0.01	0.08 ± 0.01*
Dec	0.25 ± 0.02	0.07 ± 0.01*	0.26 ± 0.01	0.08 ± 0.01*	0.25 ± 0.01	0.08 ± 0.01*

Symbol ‘*’ indicates significant differences (*P* < 0.05) between the controlled and drought-simulation groups of each tree species in the same month.

### Growth patterns of DBH, tree height, crown area, and LAI of all the groups

**Figure 3:**
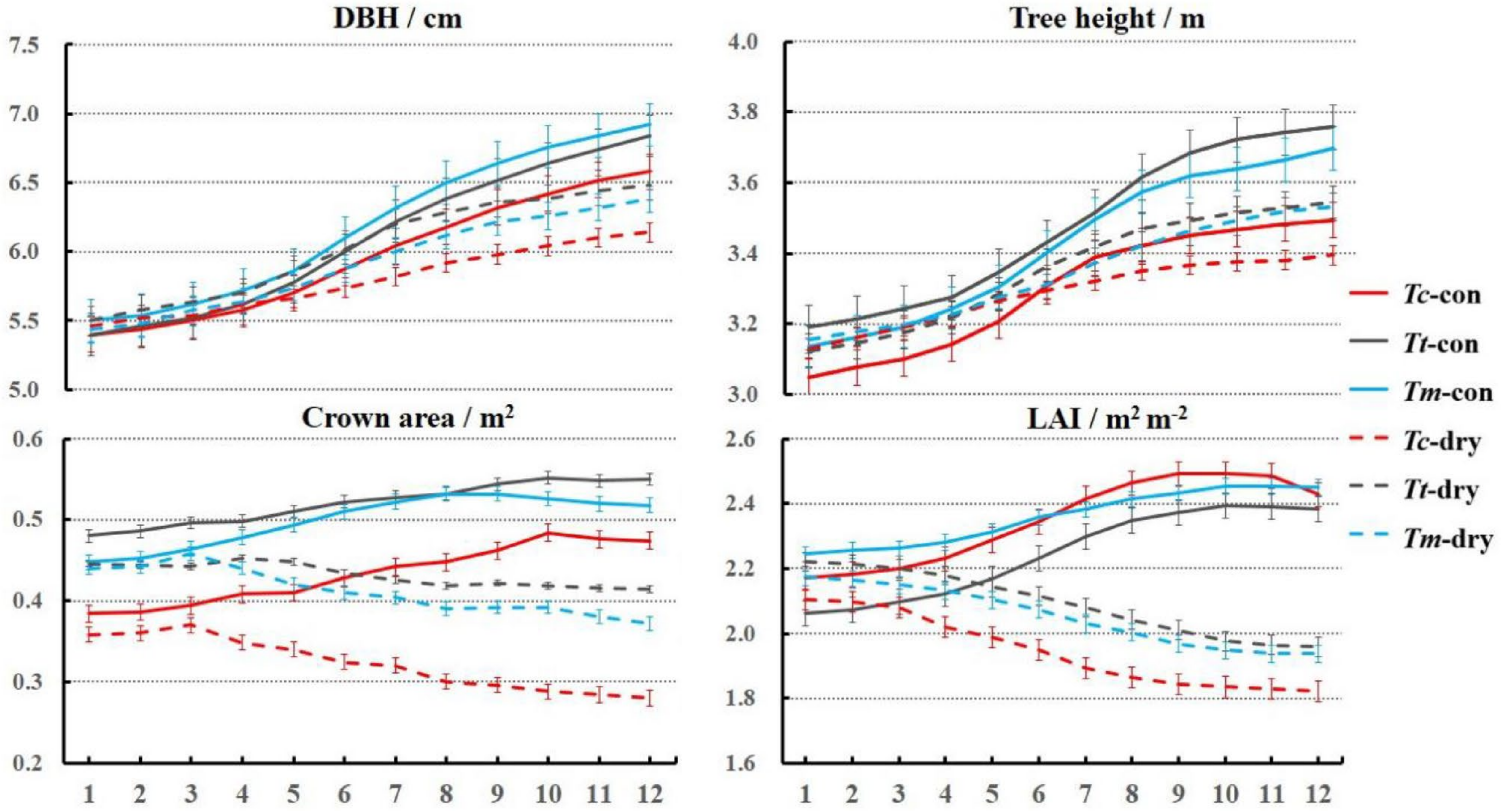
Monthly development of tree height (m), DBH (cm), crown area (m^2^), and LAI with standard deviations of *Tc* (red), *Tt* (cyan), and *Tm* (blue) in both the control (full line) and drought-simulation groups (dotted line) from January to December in 2020.

Figure 3 gave the above-ground growth of the three surveyed tree species from both the controlled and drought-simulation groups within the whole year 2020. Basically, in the controlled environment, differences in tree growth existed among the three tree species mainly due to their own characteristics. However, these differences were amplified in the drought-simulation groups that drought stress affected their growth patterns remarkably.

Concerning DBH and tree height, the three tree species displayed steady development in the controlled groups within the whole year (e.g., DBH: *Tc*-con: 5.40 ± 0.16 cm to 6.58 ± 0.13 cm; Tree height: *Tt*-con: 3.19 ± 0.09 m to 3.76 ± 0.14 m). Besides, the growth patterns were observed great acceleration from May to August (e.g., DBH: *Tm*-con: from 5.86 ± 0.11 cm to 6.50 ± 0.21 cm; Tree height: *Tc*-con: from 3.21 ± 0.15 m to 3.42 ± 0.10 m; *Tt*-con: from 3.35 ± 0.10 m to 3.62 ± 0.12 m). In the drought-simulation groups, their growth was severely weakened that significantly lower values of DBH and tree height were observed in December (e.g., DBH: *Tc*-con: 6.58 ± 0.13 cm and *Tt*-dry: 6.14 ± 0.15 cm; *Tm*-con: 6.92 ± 0.13 cm and *Tm*-dry: 6.38 ± 0.13 cm; Tree height: *Tt*-con: 3.76 ± 0.14 m and *Tt*-dry: 3.54 ± 0.11 m).

In terms of crown development, the effect caused by drought led to more distinctly reductions of the drought-simulation groups in comparison to the controlled groups of the three tree species (*P* < 0.05). In the controlled groups, the three tree species increased their crown area for several months (e.g., *Tc*-con: from 0.38 ± 0.08 m^2^ in January to 0.48 ± 0.10 m^2^ in October; *Tm*-con: from 0.45 ± 0.04 m^2^ in January to 0.53 ± 0.05 m^2^ in September), together with which their LAI had similar development tendencies (e.g., *Tt*-con: from 2.06 ± 0.11 m^2^ m^-2^ in January to 2.39 ± 0.14 m^2^ m^-2^ in October). However, both the two parameters stopped growth or declined slightly after October (e.g., crown area of *Tt*-con: 0.55 ± 0.07 m^2^ in October and 0.55 ± 0.08 m^2^ in December; LAI of *Tc*-con: 2.49 ± 0.22 m^2^ m^-2^ in October and 2.43 ± 0.28 m^2^ m^-2^ in December). On the contrary, the drought-simulation groups showed general descending trends. For the crown area, their decreases mainly started from March (e.g., *Tm*-dry: 0.44 ± 0.03 m^2^ in January; 0.45 ± 0.04 m^2^ in March; and 0.37 ± 0.02 m^2^ in December), while their LAI showed continuous decreases for the whole year (*Tc*-dry: from 2.10 ± 0.18 m^2^ m^-2^ to 1.82 ± 0.15 m^2^ m^-2^; *Tt*-dry: from 2.22 ± 0.08 m^2^ m^-2^ to 1.96 ± 0.05 m^2^ m^-2^; *Tm*-dry: from 2.17 ± 0.12 m^2^ m^-2^ to 1.94 ± 0.15 m^2^ m^-2^).

### Dynamic development of fine root biomass of all the groups

The development of the overall fine root biomass of the three tree species from the controlled and drought-simulation groups was given in Table 2 below. Under common circumstances, the three tree species had substantial growth in 2020, especially the period from May to September (e.g., *Tm*-con: 93.02 ± 28.16 g m^-2^ in May, 137.97 ± 44.92 g m^-2^ in September). From September to December, the growth of *Tt*-con and *Tc*-con was almost stagnant (*Tt*-con: from 188.48 ± 67.95 g m^-2^ to 189.59 ± 64.04 g m^-2^; *Tc*-con: from 160.95 ± 50.24 g m^-2^ to 156.41 ± 51.91 g m^-2^), while *Tm*-con remained to increase from 137.97 ± 44.92 g m^-2^ to 161.70 ± 47.78 g m^-2^. In the drought-simulation groups, the three tree species showed different patterns, though the drought had enormously restrained their growth compared to their controlled groups. *Tc*-dry, the only tree species with perpetual loss of root biomass, decreased from 64.79 ± 18.11 g m^-2^ to 28.24 ± 12.66 g m^-2^ since PEI was installed. For *Tm*-dry, it maintained its fine root biomass in the first nine months (February: 70.65 ± 9.06 g m^-2^; May: 73.09 ± 20.31 g m^-2^; September: 73.11 ± 28.36 g m^-2^) and decreased to 57.31 ± 21.78 g m^-2^ in the last three months. *Tt*-dry was the unique group that had obvious development under drought stress (February: 48.71 ± 17.27 g m^-2^; September: 78.32 ± 33.78 g m^-2^). Although it decreased to 69.26 ± 32.83 g m^-2^ from September to December, the fine root biomass was still higher than that in February, which showed a robust growth within the whole year.

**Table 2:** Overall fine root biomass (g m^-2^) with standard deviations of *Tc*, *Tt*, and *Tm* from the controlled and drought-simulation groups from January to December in 2020.

Tree species	Groups	Months			
		Feb	May	Sep	Dec
*Tc*	*Tc*-con	68.67 ± 22.76	117.78 ± 33.84	160.95 ± 50.24	156.41 ± 51.91
	*Tc*-dry	64.79 ± 18.11	50.07 ± 27.79	31.70 ± 12.47	28.24 ± 12.66
*Tt*	*Tt*-con	58.07 ± 10.07	137.74 ± 59.63	188.48 ± 67.95	189.59 ± 64.04
	*Tt*-dry	48.71 ± 17.27	52.73 ± 25.84	78.32 ± 33.78	69.26 ± 32.83
*Tm*	*Tm*-con	62.48 ± 26.78	93.02 ± 28.16	137.97 ± 44.92	161.70 ± 47.78
	*Tm*-dry	70.65 ± 9.06	73.09 ± 20.31	73.11 ± 28.36	57.31 ± 21.78

**Figure 4:**
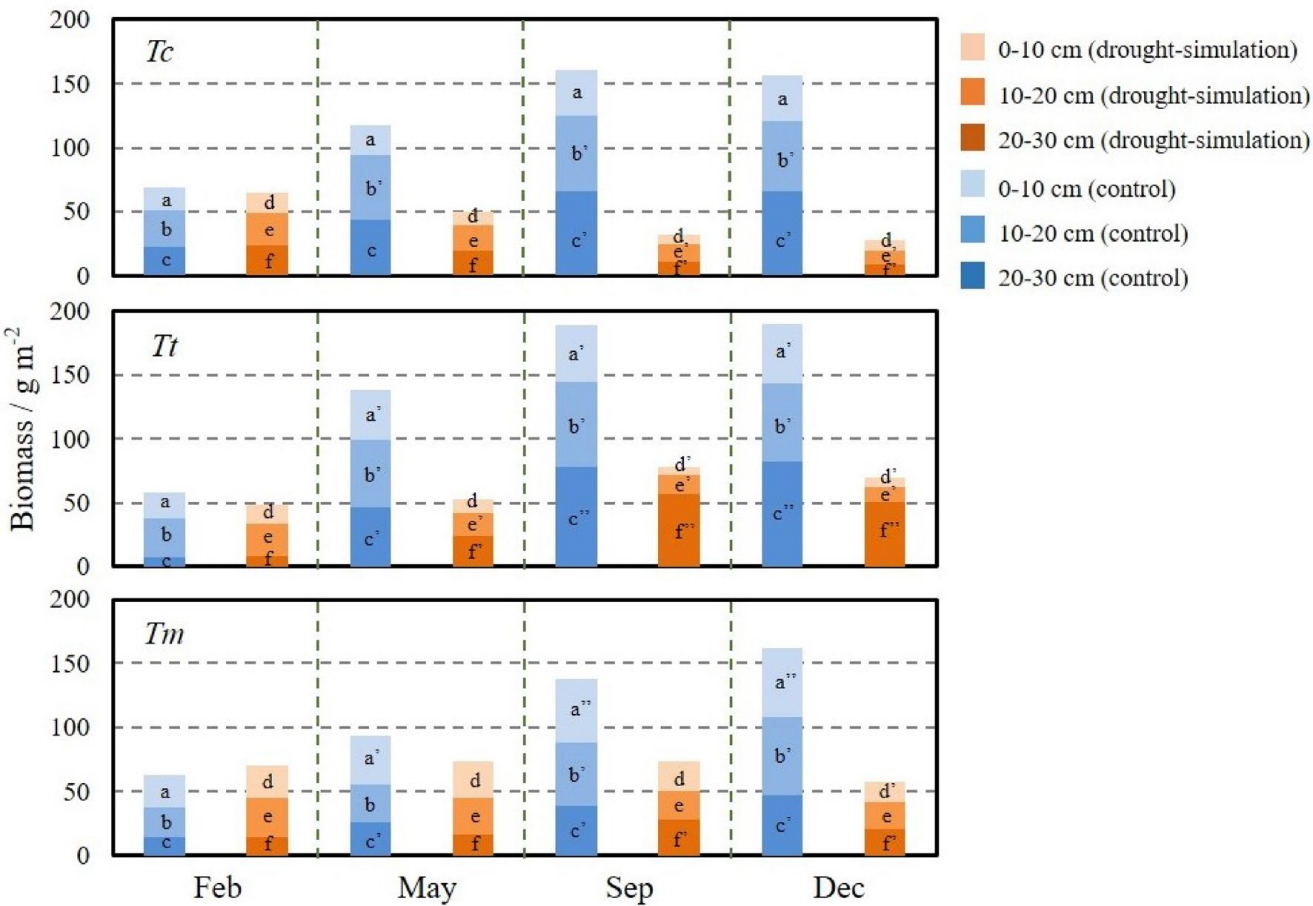
Vertical development of fine root biomass (g m^-2^) of *Tc*, *Tt*, and *Tm* from January to December in 2020. The bars in different shades of orange and blue represent the fine roots in the soil layer of 0–10 cm, 10–20 cm, and 20–30 cm from the drought-simulation groups, respectively. The different lowercase letters indicate significant differences (*P* < 0.05) performed by ANOVA with HSD test on each layer in different months.

For insights into the fine root biomass development at the vertical level, Fig. 4 gave the details for both the controlled and drought-simulation groups. The controlled groups of the three tree species increased considerably, while their drought-simulation groups exhibited different evolution patterns.

For *Tc*, *Tc*-con had distinct growth, such as the deep layer increasing from 23.02 ± 9.55 g m^-2^ in February to 65.79 ± 19.72 g m^-2^ in September. However, the three layers of *Tc*-dry displayed complete recessions in 2020 (shallow layer: from 16.38 ± 4.98 g m^-2^ to 8.80 ± 3.18 g m^-2^; middle layer: from 25.02 ± 7.33 g m^-2^ to 10.94 ± 3.56 g m^-2^; deep layer: from 23.40 ± 8.45 g m^-2^ to 8.51 ± 4.01 g m^-2^). On the contrary, though not higher than *Tt*-con, *Tt*-dry had robust development particularly for its deep layer from May to September (from 24.59 ± 9.16 g m^-2^ to 56.72 ± 23.61 g m^-2^, *P* < 0.05). From September to November, it remained to reach 51.10 ± 17.23 g m^-2^, which was the highest value of all the drought-simulation groups. Concerning *Tm*’s fine root growth at three layers, its controlled group displayed steady increments within the whole year (shallow layer: from 24.71 ± 8.22 g m^-2^ to 53.67 ± 21.05 g m^-2^; middle layer: from 23.10 ± 10.53 g m^-2^ to 61.29 ± 24.71 g m^-2^; deep layer: from 14.67 ± 5.83 g m^-2^ to 46.74 ± 17.66 g m^-2^). Nevertheless, the three layers of *Tm*-dry showed slight fluctuations from February to September. Its noticeable decreases occurred for its shallow and deep layers from September to December, which declined from 22.74 ± 9.99 g m^-2^ and 27.52 ± 12.12 g m^-2^ to 15.84 ± 6.23 g m^-2^ and 20.62 ± 8.74 g m^-2^.

**Figure 5:**
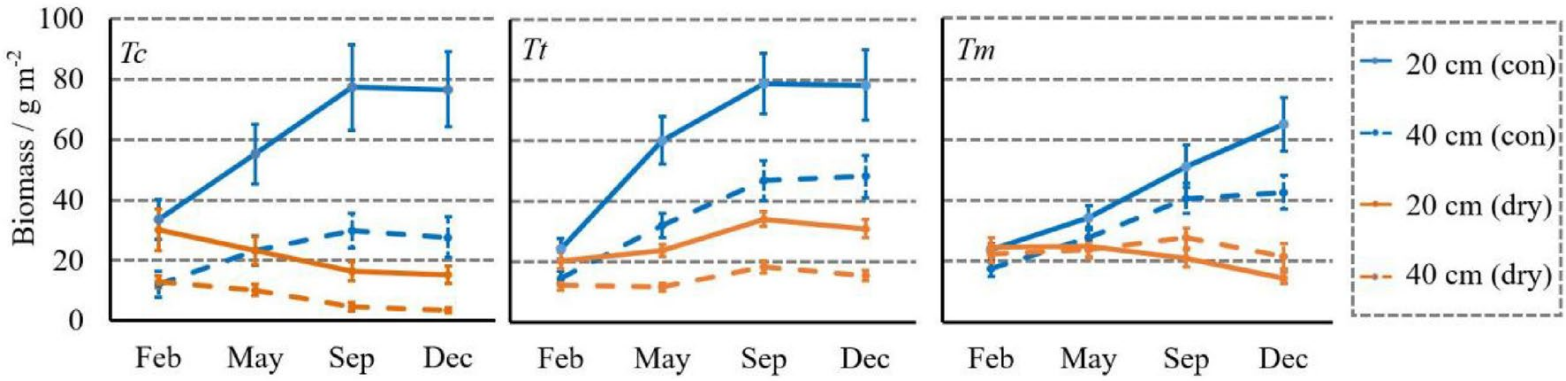
Horizontal development of fine root biomass (g m^-2^) of *Tc*, *Tt*, and *Tm* with standard deviation from January to December in 2020. The blue lines and blue dashed lines represent the fine roots at 20 cm (paracentral roots) and 40 cm (outer roots) to tree trunks in the controlled groups, while orange lines and orange dashed lines reflect those in the drought-simulation groups.

The details of root biomass development at horizontal levels were shown in Fig. 5, indicating the three tree species had different biomass allocations between their paracentral and outer roots. For *Tc*, its paracentral root biomass growth was higher than that of outer roots in the controlled group since May (e.g., paracentral root biomass in September: 77.33 ± 28.24 g m^-2^; outer root biomass in September: 29.97 ± 11.36 g m^-2^). Under drought stress, both its paracentral and outer root biomass decreased from February to December. Regarding *Tt*, the growth patterns of its paracentral and outer roots were extremely synchronous, which was mainly different in their change extents. For example, from September to December, the root biomass in the controlled group was stationary (paracentral root biomass: from 78.84 ± 19.94 g m^-2^ to 78.29 ± 23.18 g m^-2^; outer root biomass: from 46.81 ± 12.84 g m^-2^ to 48.10 ± 13.98 g m^-2^), while the drought-simulation group exhibited decreases (paracentral root biomass: from 34.07 ± 4.64 g m^-2^ to 30.82 ± 6.02 g m^-2^; outer root biomass: from 18.14 ± 4.06 g m^-2^ to 15.34 ± 3.68 g m^-2^). Concerning *Tm*’s horizontal root growth, the allocation was more average in the controlled group that the difference between its paracentral root and outer roots was much less than that of *Tc*-con and *Tt*-con. However, the allocation was inclined to outer roots in the drought-simulation group. From May to September, its paracentral root biomass decreased from 24.91 ± 6.96 g m^-2^ to 21.03 ± 5.72 g m^-2^, while the out root biomass rose from 23.82 ± 5.52 g m^-2^ to 27.71 ± 6.46 g m^-2^. After three months’ decline coming to December, the paracentral root biomass of 14.54 ± 3.56 g m^-2^ remained to be lower than the outer root biomass of 21.67 ± 8.42 g m^-2^.

### Simulated ecosystem services of all the groups

**Figure 6:**
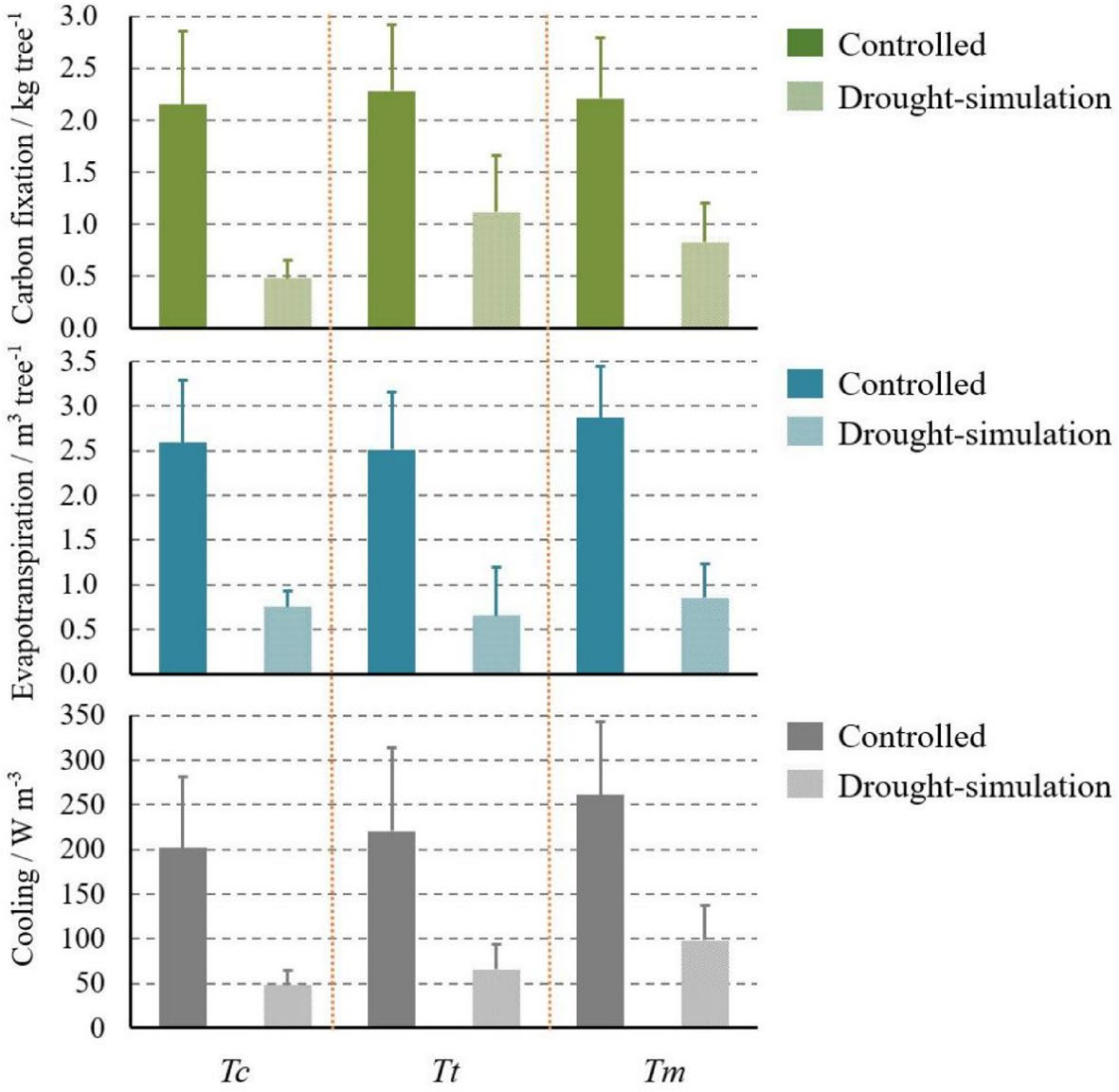
Values of simulated ecosystem services with standard deviation, including carbon fixation (kg tree^-1^), evapotranspiration (m^3^ tree^-1^), and cooling effect (W m^-3^) of *Tc*, *Tt*, and *Tm* from the controlled and drought-simulation groups at the end of 2020.

The simulated ecosystem services of three tree species were compared for their controlled and drought-simulation groups in Fig. 6. At the end of 2020, the functions of carbon fixation, evapotranspiration, and cooling of the three surveyed tree species were severely reduced under drought stress (*P* < 0.05). In terms of carbon fixation, *Tc*-dry experienced the most serious decrease in comparison to *Tc*-con (*Tc*-con: 2.15 ± 0.70 kg tree^-1^; *Tc*-dry: 0.48 ± 0.18 kg tree^-1^). On the contrary, *Tt*-dry showed the least amount of carbon loss among the three tree species which reached 49.13% of *Tt*-con (*Tt*-con: 2.28 ± 0.64 kg tree^-1^; *Tt*-dry: 1.12 ± 0.54 kg tree^-1^). Conversely, *Tt* showed the weakest evapotranspiration ability among the three tree species (*Tt*-con: 2.51 ± 1.10 m^3^ tree^-1^; *Tt*-dry: 0.65 ± 0.28 m^3^ tree^-1^), while both *Tm* exhibited the highest values (*Tm*-con: 2.87 ± 1.02 m^3^ tree^-1^; *Tm*-dry: 0.85 ± 0.38 m^3^ tree^-1^). Similarly, *Tm* displayed the strongest capacity for cooling (*Tm*-con: 260.75 ± 82.76 W m^-3^; *Tm*-dry: 98.12 ± 39.7 W m^-3^), following *Tt*’s moderate ones (*Tm*-con: 220.15 ± 93.5 W m^-3^; *Tm*-dry: 65.72 ± 27.76 W m^-3^). Concerning *Tc*’s cooling, not only *Tc*-con behaved worst among the three tree species (*Tc*-con: 201.78 ± 78.96 W m^-3^), but also *Tc*-dry was impaired by the drought effect ulteriorly (*Tc*-dry: 47.89 ± 16.52 W m^-3^).

## Discussion

### Above-ground development patterns in water shortage

The above-ground development of urban trees was tightly related to their physiological conditions and multiple ecosystem services^26,27^. Furthermore, tree species might adjust their above-ground patterns under various environmental challenges and stresses^28^. In this study, with the set of the controlled and drought-simulation groups, the three tree species exhibited different above-ground dynamic variations for their DBH, tree height, and crown areas. With similar initial statuses in the common environment, *Tm* showed distinct advantages in DBH and *Tt* had the highest values of tree height and crown area, while *Tc*’s above-ground growth was relatively flat, which was also proved in other research^29,30^. In the drought-simulation groups, the above-ground growth patterns of the three tree species were affected to various extents, of which the most drastic decrease was found for the crown area and LAI. Among the three tree species, *Tc* had the greatest recession of its crown area and LAI, which was consistent with some previous research that *Tc* was hard to maintain its canopy in water shortage^31^. As reported, the crown of urban cities played an essential role in providing multiple ecosystem services^32,33^. This was because trees’ crowns were deeply involved with their transpiration processes and creating recreational spaces, which made immense contributions to the cooling effect and landscape demands^34–36^. Based on our findings regarding the above-ground patterns, it could be inferred that *Tc* suffered most severely from the drought effect, which most probably resulted in its failure in providing basic ecosystem services, such as mitigating heat island effect and improving landscape aesthetics.

### Strategies of fine root development at both vertical and horizontal levels

As the primary pathway for water and nutrient uptake, fine roots played a key role in maintaining tree growth, which was tightly related to multiple ecosystem service^37,38^. Besides, the below-ground development patterns could adequately reflect urban trees’ investment strategies and responses to various disturbances^39,40^.

Under undisturbed circumstances in this study, the three tree species had stable overall fine root biomass growth, which started to accelerate in May and slowed down since September. On the contrary, due to the installation of the PEI, the fine root biomass growth in the drought-simulation groups was impacted severely. Among the three tree species, *Tc* was the only tree species that had a continuous decrease of overall fine root biomass, which was consistent with other research in German cities^20^. Combined with the high average temperature and major precipitation in June and August, *Tc*’s fine root biomass exhibited a significant reduction from May to September, indicating its impaired root system and inactive below-ground growth. As a comparison, *Tt* and *Tm* showed more positive fine root growth, especially for *Tt*, it remained to maintain the biomass increase, though less than the amplification in its controlled group. Considering it originating from dry regions such as southeast Europe, it could adapt to water shortage better than common urban tree species^31^.

In addition to the analysis of the overall biomass of the three tree species, their detailed development in different directions was also evaluated for further insights into how they made responses to drought stresses. As some researchers reported, enhancing deep roots could be crucial approaches to alleviate water shortage^41,42^, especially in tropical and subtropical environments^43^. This was because the construction of shallow roots required less energy, and the soil close to the ground-surface could provide more water from precipitation to support root growth generally^44^. Nevertheless, the drought effect resulting from exclusion of rainfall might lead to the headmost arid environment of upper soil layer, while the deep soil layer remained moist. In this way, many drought-tolerant tree species would invest more in their deep roots to seek for more water^20^. In this research, *Tt*-dry had the most distinct growth for its fine root at the deep layer, which was thought to be a positive reaction to deal with drought stress. The steady patterns of fine root biomass of *Tm*-dry also proved a continuous investment under water deficit, which could remained to be thought as positive indication against drought stress. Among the three tree species, *Tc*-dry was observed to have the unique drastic reduction of deep root biomass, which was also verified in some pervious research^45^. Combined with its remarkable decrease of the overall biomass, it suggested that *Tc* did not made positive reaction to drought stress during the whole year, leading to a weak capacity of coping with the arid environment.

Apart from the vertical development, the horizontal growth of fine root biomass might reflect trees’ adaption to drought stress from another perspective. For *Tc* and *Tt*, the simulated drought affected but not reversed the growth patterns completely, that the paracentral and outer root biomass of *Tc*-dry and *Tt*-dry was merely lower than the ones of *Tc*-con and *Tt*-con. This findings was similar to other researchers’ results that the root systems of *Tc* and *Tt* were stem-centered and they did not have inclinations for roots at horizontal levels facing drought stress^31^. On the contrary, *Tm*-dry was observed to increase its outer roots than paracentral roots, which was thoroughly opposite to the patterns of *Tm*-con. Combined with the little fluctuation of its vertical root biomass, this could be the particular strategy of *Tm* to cope with drought stress through the expansion of root system.

To be concluded, though it was hard to maintain the common growth under drought stress than in the common environment, drought-tolerant tree species would adjust their root development to enhance water uptake, e.g., deep root growth or constructions on horizontal roots. Among the three tree species, *Tc* showed the least evidence in root development, which was worried for its survival when drought events occurred.

### Simulated ecosystem services under drought stress

Urban trees could provide multiple ecosystem services to benefit human beings, however, these services showed diverse variability under various environmental challenges^46,47^. In this research, the carbon fixation, evapotranspiration, and cooling showed significant but different decrease under drought stress, implying the three tree species had different adaption to arid environments. Regarding carbon fixation, *Tc*-dry had the highest values of reduction compared to *Tc*-con. Combined with its above- and below-ground growth mentioned above, the shrinkage of tree growth could account for the loss of carbon fixation adequately. To the opposite, the vigorous growth of *Tt*-dry, specifically for its fine root development, could be the pivotal importance for its relatively higher carbon fixation than the other two tree species. Concerning the functions of evapotranspiration and cooling, the three tree species showed great reductions as well, of which *Tm* exhibited the highest values in the controlled and drought-simulation groups. As a native tree species in Guangzhou for several years, *Tm* could be more adaptive in hot environment, which probably led to its maintenance of ecosystem services. In details of *Tm* and *Tc*, the decrease of cooling function of *Tm*-dry might be explained by its inclination to biomass storage and root growth, whereas that reduction of *Tc*-dry could result from the tree growth recession caused by its weak drought tolerance.

### Appropriate selection of urban tree species in Guangzhou

The selection of urban tree species for urban greening should cautiously take tree’s adaption to the local environment into account^45,48^. Meanwhile, considering the surge of global change, how well trees responded to extreme climatic events should be profoundly evaluated for their conservation and management in the future^22^. In cities such as Guangzhou with rapid increasing urbanization together with a trend of rising temperature and reduced precipitation, the introduced or selected urban tree species were better to cope with drought positively to avoid high mortality together with economic loss. *Tm*, as a native tree species, did not show enormous failure in tree growth and ecosystem services in water shortage, indicating its well adaption in the local regions. As the two potential introduced tree species, *Tc* and *Tt* behaved quite differently under arid circumstances. For *Tc*, its seriously impacted above- and below-ground growth demonstrated its unsatisfied adaption to drought stress, whereas *Tt*’s root development from deep layer could be positive investment to mitigate water deficit. Moreover, *Tt*-dry’s carbon fixation demonstrated that it could survive better than *Tc* facing lack of water. Hence, considering the increasing intensities and frequencies of drought events in the future, *Tt* could be a better section than *Tc* in Guangzhou. If *Tc* was introduced, special management including water supply should be adopted to promote its growth and ecosystem services.

## Conclusion

Generally, cities might launch introduction campaigns of exotic tree species to meet various requirements, e.g., constructions of urban greening, improvement of regional landscape values, and promote local diversities. On this basis, the campaign should cautiously consider the adaption of introduced tree species due to the local environments. In Guangzhou, the trend of increasing temperature with reduced precipitation had been verified. In this way, a drought-simulation experiment was launched to evaluate the growth and ecosystem services of the two newly-introduced tree species *Tc* and *Tt* in comparison to the native *Tm*.

Under water shortage, the three surveyed tree species showed decreases of tree growth and ecosystem services to different extents. *Tm*, as a native tree species, exhibited moderate patterns of growth and advantages in evapotranspiration and cooling. Besides, its investment in root development at horizontal level could account for its special strategy against drought stress. In terms of *Tt*, its vigorous root growth could be the most positive behave of coping with water deficit, which explained its maintenance of carbon fixation and implied a well adaption. Among the three tree species, *Tc* showed complete decrease of above- and below-ground growth, especially for its fine root biomass. In addition, its ecosystem services were significantly reduced, reflecting a comprehensive failure when it faced long-term scarcity of water. Therefore, *Tt* might adapt to potential drought events in the future better than *Tc*. If they are planted in Guangzhou, it was necessary to supply adequate water and below-ground space for them, especially for *Tc*. To amplify their multiple ecosystem services, long-time observation on their growth under different stresses can be effective approaches in the future.

## Data Availability

The datasets generated and/or analyzed during the current study are available from the corresponding authors on reasonable request.
